# Flash Glucose Monitoring: A Review of the Literature with a Special Focus on Type 1 Diabetes

**DOI:** 10.3390/nu10080992

**Published:** 2018-07-29

**Authors:** Giulia Mancini, Maria Giulia Berioli, Elisa Santi, Francesco Rogari, Giada Toni, Giorgia Tascini, Roberta Crispoldi, Giulia Ceccarini, Susanna Esposito

**Affiliations:** Pediatric Clinic, Department of Surgical and Biomedical Sciences, Università degli Studi di Perugia, 06132 Perugia, Italy; giu87manci@gmail.com (G.M.); mgiuliaberioli@gmail.com (M.G.B.); elisa.santi1988@gmail.com (E.S.); rogari.francesco@virgilio.it (F.R.); toni.giada@gmail.com (G.T.); giorgia.tascini@gmail.com (G.T.); roberta.crispoldi@yahoo.it (R.C.); giulia.cec@hotmail.it (G.C.)

**Keywords:** continuous glucose monitoring, flash glucose monitoring, type 1 diabetes

## Abstract

In people with type 1 diabetes mellitus (T1DM), obtaining good glycemic control is essential to reduce the risk of acute and chronic complications. Frequent glucose monitoring allows the adjustment of insulin therapy to improve metabolic control with near-normal blood glucose concentrations. The recent development of innovative technological devices for the management of T1DM provides new opportunities for patients and health care professionals to improve glycemic control and quality of life. Currently, in addition to traditional self-monitoring of blood glucose (SMBG) through a glucometer, there are new strategies to measure glucose levels, including the detection of interstitial glucose through Continuous Glucose Monitoring (iCGM) or Flash Glucose Monitoring (FGM). In this review, we analyze current evidence on the efficacy and safety of FGM, with a special focus on T1DM. FGM is an effective tool with great potential for the management of T1DM both in the pediatric and adult population that can help patients to improve metabolic control and quality of life. Although FGM might not be included in the development of an artificial pancreas and some models of iCGM are more accurate than FGM and preferable in some specific situations, FGM represents a cheaper and valid alternative for selected patients. In fact, FGM provides significantly more data than the intermittent results obtained by SMBG, which may not capture intervals of extreme variability or nocturnal events. With the help of a log related to insulin doses, meal intake, physical activity and stress factors, people can achieve the full benefits of FGM and work together with health care professionals to act upon the information provided by the sensor. The graphs and trends available with FGM better allow an understanding of how different factors (e.g., physical activity, diet) impact glycemic control, consequently motivating patients to take charge of their health.

## 1. Introduction 

Several studies have widely demonstrated that elevated levels of glycated hemoglobin (HbA1c) lead to long-term microvascular and macrovascular complications [1,2]. Therefore, in people with type 1 diabetes mellitus (T1DM), obtaining good glycemic control is essential to reduce the risk of acute and chronic complications [1,2]. Frequent glucose monitoring allows the adjustment of insulin therapy to improve metabolic control with near-normal blood glucose concentrations [3,4,5]. The recent development of innovative technological devices for the management of T1DM provides new opportunities for patients and health care professionals to improve glycemic control and quality of life [6,7,8]. Currently, in addition to traditional self-monitoring of blood glucose (SMBG) through a glucometer, there are new strategies to measure glucose levels, including the detection of interstitial glucose through Continuous Glucose Monitoring (iCGM) or Flash Glucose Monitoring (FGM) [3,4,5,6,7,8]. In this review, we will analyze current evidence on the efficacy and safety of FGM, with a special focus on T1DM.

## 2. Current Strategies to Detect Glucose Levels

The advent of new technological devices has provided new options to measure glucose concentrations. While conventional glucometers determine blood glucose (SMBG), sensors for iCGM and FGM detect interstitial glucose levels. 

SMBG has many limitations, including insufficient identification of glycemic variability and hyperglycemic or hypoglycemic episodes due to intermittent monitoring, unreliability of patient recorded data and inadequate compliance due to pain and discomfort associated with fingerstick capillary blood sampling.

Commercially available iCGM systems were introduced in 2000 [3], and the first models were retrospective, meaning that data were available only at the end of the sensor wear time. Real-time iCGM (RT-iCGM) was then developed, showing glucose levels in real time (values are automatically displayed every 1–5 min), as well as their rate of change and glucose trends. However, there is a “lag time” between the plasma and interstitial fluid; therefore, interstitial glucose values do not correspond exactly to blood glucose concentration, which may cause a loss of accuracy for RT-iCGM, especially during rapid glycemic excursions [3]. To maintain accurate sensor glucose readings, iCGM systems require daily capillary blood calibrations (typically twice daily at stable glucose values). At present, only the Dexcom G6 iCGM system does not require fingersticks for calibration, and only the Dexcom G6 and G5 iCGM systems can replace SMBG for insulin-dosing decisions [4]; the other types of RT-iCGM are approved as an adjunct to SMBG [5]. In real-time iCGM systems, alarms can be programmed in case glycemic values are below or above a target range. This feature is especially useful to detect unsuspected hypoglycemia (such as during sleep). Furthermore, RT-iCGM can be associated with Continuous Subcutaneous Insulin Infusion (CSII), thus obtaining a Sensor-Augmented Pump (SAP), which in some cases includes algorithms capable of interrupting insulin infusion when the glucose concentration reaches or is expected to reach a defined level. These options, which significantly help to prevent hypoglycemia, are only available with iCGM and are an important step towards automated closed-loop systems and an artificial pancreas. 

FreeStyle Libre, the first FGM system, was brought to the market in Autumn 2014 and represents a new option in glucose monitoring [5] that is cheaper than available iCGM systems [6,7]. The FreeStyle Libre system uses a wired glucose oxidase enzyme co-immobilized on an electrochemical sensor that is worn on the arm for up to 14 days [8]. This type of patch sensor is about the size of a coin and has a short filament (4 mm long) that must be inserted into the subcutaneous tissue of the upper arm. The main differences between iCGM and FGM sensors are summarized in Table 1. In contrast to iCGM, the FGM system does not require calibration, thanks to a technology that allows a factory calibration [5]. The main advantages of factory calibration compared to user calibration are elimination of fingersticks required for calibration and avoiding potential errors during the calibration process (e.g., an unsuitable moment, incorrect reference measurement due to failure of the blood glucometer, contaminated skin), which can lead to sensor inaccuracies and are one of the principal sources of sensor errors [3]. Another feature that distinguishes FGM from iCGM is the availability of glucose data only on demand; in FGM, the glucose values are not constantly shown, and people can obtain real-time interstitial glucose values by placing a “reader” in proximity to the sensor. Data are transferred from the sensor to the reader and recorded automatically every 15 min; in addition, trends for the previous 8 h can be seen on the screen [8]. Similar to iCGM systems, the glucose change trend is indicated using an arrow, but in contrast to iCGM, there are no alarms when defined values are exceeded or expected to be exceeded in the following minutes. Further, FGM lacks connectivity with CSII devices. Glucose values, which can be downloaded at any time, are presented in a simple form and include the Ambulatory Glucose Profile (AGP), which combines all the data from the sensor over a period of 14 days and gives a summarized visual display of glycemic patterns. 

## 3. Accuracy of Flash Glucose Monitoring (FGM)

Several studies have assessed the accuracy of the FreeStyle Libre system using different methods and parameters and involving different populations. In general, FGM resulted in acceptable or good accuracy both in adult and in pediatric populations with diabetes [9,10,11,12,13,14]. The site of insertion of FGM seems to be important for its accuracy; a study [7] demonstrated acceptable accuracy for the FGM readings in the upper arm, while data obtained from the abdomen were not reliable. With regard to lag time, Ji et al. [9] reported a mean lag time between the sensor and the venous Yellow Springs Instrument (YSI) reference of 3.1 min, while Bailey et al. [14] observed a mean lag time of 4.5–4.8 min when comparing FGM to the same reference method.

With regard to the mean absolute relative difference (MARD), Bailey et al. [14] reported good accuracy of FGM compared with that of capillary blood glucose in patients with T1DM, with an overall MARD of 11.4%. Accuracy remained stable over 14 days of wear and was not affected by patient characteristics, such as body mass index, age, clinical site, insulin administration, or HbA1c. Other studies found a slightly different MARD when comparing FGM to different methods (capillary blood glucose, arterial blood glucose, venous YSI, laboratory random blood sugar). In most cases, MARD varied from 9.56% to 15.4% [9,10,11,12,15,16,17]. Some authors reported a higher MARD; Sekido et al. [18] observed a value of 17.1% (compared to plasma glucose), Massa et al. observed a value of 16.7% (compared to capillary blood glucose) [19], while Schierenbeck [20] found a poorer performance of the system, with an overall MARD of 30.5% (compared to arterial blood glucose). In the research by Ancona et al. [13], MARD was lower (14%) when FGM was compared to arterial blood glucose than when FGM was compared to capillary blood glucose (MARD 20%), but this difference could be explained by particular features of the critically ill patients analyzed (e.g., a level of hematocrit below normal). Some studies showed that accuracy remained stable throughout 14 days of use [3,9,10,14], while Freckmann et al. [21] highlighted a higher MARD on the first day of use compared to on days 2–14. Similarly, Ji et al. [9] reported a higher MARD during the first 9 h after sensor insertion than in the following days. Accuracy seems to be lower in lower glucose ranges [7,10,11,15,17,20,22].

With regard to the discrepancy between FGM measurements and the reference method, some authors reported FGM values lower than those in arterial, [13,20] capillary [10,13] or venous blood [22]. In the study by Ancona et al., this result may have been influenced by factors related to the impairment of blood supply and glucose diffusion in the critically ill patients who were analyzed. Fokkert [7] observed lower values with FGM in the lower glucose ranges and an underestimation of the effect of a meal on glucose response, while Sekido et al. [18] observed higher values with FGM than plasma glucose during an oral glucose tolerance test (OGTT) in healthy volunteers.

Some authors evaluated the accuracy of FGM by comparing it to different kinds of iCGM systems. Two studies [15,17] found good agreement between FreeStyle Libre and Dexcom G4 Platinum (DG4P) in adult people with T1DM; in particular, Boscari et al. [17] observed a better accuracy of FreeStyle Libre during moderate and rapid glucose changes, while Bonora et al. [15] showed a decrease in agreement in the last four days. Similarly, Aberer [22] compared the FreeStyle Libre, DG4P and Medtronic MiniMed 640G systems in people with T1DM and found a superior accuracy of the Abbott system in all glycemic ranges and during exercise.

There are not many studies about FGM that are specific to the pediatric population with T1DM. Edge et al. [12] demonstrated accuracy, safety and user acceptability of the FreeStyle Libre System for the pediatric population with T1DM. Sensor results versus capillary blood glucose had an acceptable accuracy, with 83.8% of results in zone A and 99.4% of results in zones A and B of the consensus error grid; MARD was 13.9%. Further, Massa et al. [19] compared FGM readings and capillary BG measurements in children, and found a reasonable agreement with an overall MARD of 16.7% and a large interindividual variability. The FGM System had a high safety and user acceptability and the usability questionnaire indicated high levels of satisfaction, despite some sensor problems mainly connected to early detachment of the sensor. The study by Hulse et al. [11] reported a good accuracy of AGP data in children with T1DM, with a lower accuracy in the lower glucose ranges (<75 mg/dL), suggesting that AGP values should be confirmed by SMBG before clinical intervention. The MARD was 9.56% for AGP over random blood sugar and 15.07% for AGP over capillary blood glucose. 

Rai et al. [23] showed that in a cohort of children with T1DM the average duration of sensor wear was 9.3 days, with the sensor remaining in situ for the complete duration of 14 days in approximately 65% of subjects. Regarding data and sensor failure, AGP was found to be a feasible option for monitoring glycemic status and was well accepted by most of the children and their parents with the exception of some due to minor discomfort. 

## 4. Efficacy and Safety of Flash Glucose Monitoring (FGM) 

Several studies have analyzed the effects of the use of FGM on metabolic control and are summarized in Table 2. The IMPACT study [24], a multicenter, randomized controlled trial involving adult patients with well controlled T1DM showed a reduced time in hypoglycemia in the intervention group using FGM compared with that in the control group using capillary strips, equating to a 38% decrease in time spent in hypoglycemia. The intervention group also showed a reduction in the time spent in hyperglycemia, an increase in time within optimum glucose control and a reduction in glycemic variability. Furthermore, HbA1c levels and insulin doses were unchanged compared to those in the control group at six months. Another study [6] performed in adult patients with T1DM found a reduction in the mean HbA1c from 8.0 ± 0.14% to 7.5 ± 0.14% after the introduction of FGM, as well as a reduction in hypoglycemic episodes during the use of the sensor. Clinical benefits of FGM use, in particular a reduction in hypoglycemia, have also been reported in patients with type 2 diabetes mellitus (T2DM) on intensive insulin therapy [25,26]. Other authors [27,28] showed improved metabolic control in both uncontrolled T2DM and T1DM people with the use of FGM. Furthermore, FGM has been positively associated with measures of quality of life and treatment satisfaction, both in adult patients with T1DM [6,10,24,27] or T2DM [26,27] and in the pediatric population [12,19].

With regard to safety, in the adult population some studies do not report severe adverse events related to the device [9,24], whereas other studies [25,26] describe some adverse events from mild to severe related to sensor-wear reactions, but not serious adverse events or severe hypoglycemic events related to sensor data use. Adverse events related to the sensor reported in the adult population include allergic reaction, moderate to severe itching, rash, erythema, edema, induration, bleeding, insertion-site symptoms, bruising, pain, minor infection at the insertion site, discomfort during insertion, the presence of blood and other fluids at the sensor site or a visible skin reaction after removal [9,10,14,24,25,29].

In the pediatric population, no severe adverse events related to the device have been reported [11,12,16,19]. Adverse reactions described in the pediatric age group include mild pain; irritation at the sensor insertion site, including itching, pressure feeling, erythema, and swelling [11,19,23]; allergic reaction, blister, pink mark/scabbing and abrasion or blood rests after removal of the sensor [12,19]; one subject with poor metabolic control had a local pustule at the removal of sensor, which healed in a couple of days without additional therapy [23].

## 5. Advantages of Flash Glucose Monitoring (FGM) in Children with Type 1 Diabetes (T1DM)

Recently, innovative technologies have revolutionized the management of T1DM. In particular, the introduction of new glucose monitoring techniques and data analysis represent an important opportunity for both patients and health care professionals and can help to alleviate the burden of T1DM management while improving the quality of life. 

The advantages of iCGM and FGM include the rapid and painless measurement of glucose levels and the possibility to generate much more information than conventional SMBG. These features appear particularly important in the pediatric age group where glycemic values can be extremely variable. These sensors thus enable a more accurate assessment of glycemic variability and the identification of nocturnal hypoglycemic episodes, consequently helping patients to achieve optimal control, reduce complications as well as improve treatment satisfaction and quality of life. Since hypoglycemic episodes can have adverse outcomes on the central nervous system, especially in children [30,31], and because glycemic variability is an independent risk factor for developing long-term complications in patients with diabetes [32,33,34,35], this is a significant achievement.

ICGM systems have some advantages over FGM, such as better accuracy in the newest models of sensors, alarms in case of hypoglycemia or hyperglycemia and the possibility to connect to an insulin pump. For example, for individuals with severe hypoglycemia unawareness, iCGM associated with predictive or low-threshold glucose insulin-suspended technology might be preferable rather than FGM [24].

However, FGM represents a good option for many patients for the following reasons: The smaller size, the ease of use and data interpretation, the lower cost versus iCGM, the absence of user calibration and the absence of a stressful alarm [29].

This review of the available literature about FGM shows the generally acceptable accuracy of this sensor both in adult and pediatric populations, with a lower accuracy in the lower glucose ranges.

With regard to safety, no severe adverse events related to the device have been reported in the pediatric population [11,12,16,19], while in the adult population, some patients have reported mild to severe adverse events for sensor-wear reactions but no serious adverse events related to sensor data use. Even if some authors have highlighted the accuracy and safety of FGM as a stand-alone system in adult patients with diabetes, similar to some models of iCGM [9,14,24], we believe that the use of FGM cannot completely replace SMBG [36]. Additional periodic blood glucose measurements are advisable to improve patient safety, especially during the first day of use, in the hypoglycemic range, during rapid glucose changes and in the case of disagreement with symptoms. In addition, Kovatchev et al. [37] stated that iCGM systems should achieve a MARD of 10% or lower compared with blood glucose values to allow reliable insulin dosing decisions based on sensor results. Although this conclusion is based on an estimate for iCGM data, it could also be applicable to FGM, whose measurement principles are similar to those for iCGM [21]. At the moment, the MARD of Freestyle Libre has been reported to be greater than 10%. With regard to the effect of FGM on metabolic control, this device could reduce hypoglycemia in both the T1DM and T2DM [6,24,25,26]. Moreover, some studies have shown a reduction in glycemic variability [24] or an improvement in the mean HbA1c^6^ with the use of FGM in the adult population with T1DM. Since the impact of FGM on metabolic control seems very promising in the adult population, further research should investigate the effect of this type of sensor on glycemic control and the frequency of hypoglycemia in pediatric patients with T1DM, as there are no current studies specific to the pediatric population on this topic.

## 6. Conclusions

FGM is an effective tool with great potential for the management of T1DM both in the pediatric and adult population that can help patients to improve metabolic control and quality of life. Although FGM might not be included in the development of an artificial pancreas due to the lack of connectivity with CSII, and although some models of iCGM are more accurate than FGM and preferable in some specific situations, FGM represents a cheaper and valid alternative for selected patients. In fact, FGM provides significantly more data than the intermittent results obtained by SMBG, which may not capture intervals of extreme variability or nocturnal events. With the help of a log related to insulin doses, meal intake, physical activity and stress factors, patients can achieve the full benefits of FGM and work together with health care professionals to act upon the information provided by the sensor. The graphs and trends available with FGM better allow an understanding of how different factors (e.g., physical activity, diet) impact glycemic control, consequently motivating patients to take charge of their health [38]. Research on the impact of FGM on glycemic control has already shown important results in adults; further studies should investigate its effect on metabolic control in the pediatric population with T1DM. Moreover, it should be clarified whether FGM should be included in an artificial pancreas to improve the control of time spent in the target glycemic range.

## Figures and Tables

**Table 1:** Comparison between Continuous Glucose (iCGM) and Flash Glucose Monitoring (FGM) systems.

System	Glucose Measurement	User Calibration	Data Display	Trend Arrows	Alarms in Case of Hypoglycemia or Hyperglycemia	Maximum Duration of Sensor	Connectivity to Insulin Pump	Adjustment of the Insulin Dose Based on Sensor Results
FGM	Interstitial	No	Showed on demand	Yes	No	14 days	No	No
iCGM	Interstitial	Yes, daily (except for Dexcom G6)	Showed automatically	Yes	Yes	Depending on the kind	Yes (not all kinds)	Yes (at the moment, only Dexcom G5 and G6)

**Table 2:** Impact of flash glucose monitoring (FGM) on metabolic control.

Authors	Population (N, Age)	DM Type	Period Analyzed	Effect on Hypoglycemia	Effect on HbA1c	Effect on Daily Insulin Dose and Oral Hypoglycemic Agents	Effect on Time in Range	Effect on Glycemic Variability	System Utilization
Dover et al. [6].	*N* = 25, mean age 39.8 ± 2.0 years	T1DM	16 weeks	reduction in the final 2 weeks of the study compared to the first 2 weeks	reduction vs. pre FGM use	n/a	n/a	n/a	n/a
Anjana et al. [28].	*N* = 2536 cases (FGM), T1DM mean age 28.0 ± 16.1 years; T2DM 57.3 ± 12.1 years	T1DM or T2DM	6 months	n/a	reduction both in cases and controls; magnitude of reduction higher among cases	Evaluated in cases vs. pre AGP use	n/a	n/a	n/a
						T1DM: insulin unchanged 18.5%, increased 46.6%, decreased 34.9%			
	*N* = 2536 controls (no FGM), T1DM mean age 27.5 ± 15.5 years, T2DM 57.1 ± 12.2 years					T2DM (not all on insulin therapy): insulin unchanged 30.4%, increased 33.2%, decreased 36.5%			
						Oral hypoglycaemic agents unchanged 71.9%, increased 20.1%, decreased 8%			
Bolinder et al. [24].	*N* = 119 cases (FGM) *N* = 120 controls (no FGM) mean age 43.7 ± 13.9 years	T1DM	6 months	reduction in the intervention vs. control group	unchanged in the intervention vs. control group	no differences between the study groups	increased in cases vs. controls	improved in the intervention vs. control group	98.8% in cases (*N* = 112)
Haak et al. [25].	*N* = 149 cases (FGM), mean age 59.0 ± 9.9 years	T2DM	6 months	reduction in the intervention vs. control group	unchanged in the intervention vs. control group Participants < 65 years: drop in HbA1c more pronounced in the intervention vs. control group	no differences between the study groups	no differences between the study groups	improved in the intervention vs. control group	88.7 ± 9.2% in cases (*N* = 138)
	*N* = 75 controls (no FGM), mean age 59.5 ± 11.0 years				Participants ≥ 65 years: drop in HbA1c more pronounced in the control vs. intervention group				
Haak et al. [26].	*N* = 139, mean age 59.3 ± 9.6 years (completed study *N* = 125)	T2DM	12 months	reduction at the end of the study compared to baseline	n/a	unchanged compared to baseline	unchanged compared to baseline	unchanged compared to baseline	88.7 ± 9.2% between 0 and 6 months, 83.6 ± 13.8% between 6 and 12 months
Ish Shalom et al. [27].	*N* = 31, mean age 58 ± 16 years	T1DM or T2DM	12 weeks	n/a	reduction compared to baseline	n/a	n/a	n/a	n/a
					patients who continued using the device (*N* = 27): the change was maintained for 24 weeks				
